# Prevalence and Antimicrobial Resistance of Thermophilic *Campylobacter* Isolated from Chicken in Côte d'Ivoire

**DOI:** 10.1155/2012/150612

**Published:** 2012-10-23

**Authors:** Goualié Gblossi Bernadette, Akpa Eric Essoh, Kakou-N'Gazoa Elise Solange, Guessennd Natalie, Bakayoko Souleymane, Niamké Lamine Sébastien, Dosso Mireille

**Affiliations:** ^1^Laboratoire de Biotechnologies, Filière Biochimie-Microbiologie, Unité de Formation et de Recherche en Biosciences, Université de Cocody-Abidjan, 01 BP 582, Abidjan, Cote d'Ivoire; ^2^Institut Pasteur de Côte d'ivoire, 01 BP 490, Abidjan, Cote d'Ivoire

## Abstract

Thermophilic *Campylobacters* are major causes of gastroenteritis in human. The main risk factor of infection is consumption of contaminated or by cross-contaminated poultry meat. In Côte d'Ivoire, gastroenteritis is usually observed but no case of human campylobacteriosis has been formally reported to date. The aims of this study were to determine prevalence and antimicrobial resistance of *Campylobacter jejuni* and *Campylobacter coli* isolated from chickens ceaca in commercial slaughter in Abidjan. Between May and November 2009, one hundred and nineteen (119) chicken caeca samples were collected and analyzed by passive filtration method followed by molecular identification (PCR). From these 119 samples, 76 (63.8%) were positive to *Campylobacter* tests. Among the positive colonies, 51.3% were *C. jejuni* and 48.7% were *C. coli*. Of the 39 *C. jejuni* isolates, 79.5%, 38.5%, 17.9%, 10.3%, and 7.7% were, respectively, resistant, to nalidixic acid, ciprofloxacin, amoxicillin, erythromycin, and gentamicin. Among the 37 isolates of *C. coli*, 78.4%, 43.2%, 13.5%, 8.1%, and 0% were resistant, respectively, to the same antibiotics. In conclusion, we reported in this study the presence of high *Campylobacter* contamination of the studied chickens. Molecular identification of the bacteria was performed and determination of high resistance to antimicrobials of the fluoroquinolone family was revealed.

## 1. Introduction

*Campylobacter bacteria* are Gram negative, curved, highly mobile, and microaerophilic. Thermophilic *Campylobacter *species, particularly *Campylobacter jejuni *and *Campylobacter coli*, have been recognized as a major cause of acute bacterial gastroenteritis in humans since 1970 and it is estimated that *Campylobacter *spp. are responsible for 400–500 millions cases of diarrhea each year worldwide [1–4]. In developing countries, incidence of children under 5 years old is estimated to 40 000 cases for 100 000 persons per year [5, 6] and according to World Health Organization (WHO) this incidence is underestimated. 

Unpasteurized milk, water, and foods of animal origin are potential sources of contaminations [7–9], but the major risk factor for campylobacteriosis for humans is the consumption of undercooked poultry and the handling of raw poultry [9–16].

Most *Campylobacter* infections do not need to be treated with antimicrobial agents. However, in a subset of patients *Campylobacter* may cause severe complications and increased risk for death and therefore requires treatment [17–19]. 

When clinical treatment is necessary, fluoroquinolones (Ciprofloxacin, e.g.) or macrolides (Erythromycin) are currently used because of their large spectra activity on enteric pathogens [20, 21].

However, antimicrobial drug resistance in *Campylobacter* infections has increased dramatically in many countries during the 1990s. This emergence of *strains* resistance to antimicrobials provoked controversy over the use of antimicrobials in animal food production [22, 23].

Because of the increasing concerns of the public regarding the risk of exposure to antibiotic-resistant bacteria through food, monitoring programs for antimicrobial resistances in indicator bacteria isolated from food animals have been developed in a number of countries [24–27].

In Côte d'Ivoire, although poultry meat and particularly broiler chicken is a major proteins source for the population, no case of human campylobacteriosis has been published to date. However, our study in 2005 [28] showed a high prevalence (67%) of *Campylobacter* spp. in chickens. This situation can be explained by the fact that *Vibrio cholerae*, *Salmonella,* and many other microorganisms, out of *Campylobacter*, are the priorities of health authorities of Côte d'Ivoire. Therefore, survey data on the prevalence of *Campylobacter* spp. mainly *C. jejuni* and *C. coli* (as they are responsible of more than 98% cases of campylobacteriosis) [29] are needed. In addition, determination of antimicrobial resistance of *Campylobacter* isolates from chickens is important. 

The aims of this study were, on one hand, to estimate prevalence of thermophilic C*ampylobacters* in slaughtered chickens in Abidjan by biochemical and molecular technical and, on the other hand, to evaluate their antimicrobial resistance.

## 2. Material and Methods 

### 2.1. Study Area and Sample Preparation

This study was conducted in Adjamé, a municipality in the north part of Abidjan. Adjamé is the principal market of Abidjan with more than 3 million visitors per day [30].

From May to November 2009, 119 samples of chicken caeca were collected in a commercial poultry processing plant. This plant is one of the primary poultry processing of this municipality with visitors of diverse origin particularly restaurateurs.

Each randomly selected sample was collected during evisceration and put into a stomacher bag, then rapidly transported to the laboratory in a cooler. 

### 2.2. Isolation and Biochemical Identification

Isolation of *Campylobacter* was performed with passive filtration method preceded by enrichment as proposed by Federighi [31]. 

Briefly, 1 g of caeca contents was transferred to 9 mL of Preston enrichment broth base (CM 0067 Oxoid, OXOID LTD., Basingstoke, Hampshire,UK) containing *Campylobacter* growth factor (SR 0232E Oxoid, OXOID LTD., Basingstoke, Hampshire, UK) and 7% (v/v) defibrinated sheep blood. Incubation was performed in an anaerobic jar containing a packet generator microaerophilic atmosphere (5% oxygen, 10% carbon dioxide, 85% nitrogen) type CAMPYGen (CN0025A Oxoid, Basingstoke, Hampshire, UK) during 24 hours at 37°C. After enrichment, 300 *μ*L of broth was filtered through acetate cellulose filter (0.45 *μ*m) on Columbia agar (Sharlau; Barcelona, Spain) containing 5% (v/v) fresh sheep blood at aerobic conditions during one hour. 

The filter was removed from agar and *Campylobacter* was isolated at 42°C during 48 hours under microaerophilic atmosphere.

One presumptive *Campylobacter* colony from each agar plate was subcultured and identified by Gram staining reaction, and biochemical pattern for oxidase, catalase, indoxyl acetate hydrolysis, and hippurate hydrolysis [32]. All isolates were stored in 25% (v/v) glycerol-peptone broth at −70°C. 

### 2.3. DNA Extraction and PCR Conditions

Genomic DNAs from all isolated colonies were obtained by treatment with dodecyl sulfate sodium and proteinase K followed by extraction with phenol-chloroform and precipitation with ethanol [33]. PCR procedures used in this study have been described previously by Linton et al. [34]. Two genes for the identification of *Campylobacter jejuni* and *Campylobacter coli* were used: *hipo* gene (encoding *C. jejuni* hippurase) and *asp* gene (encoding *C. coli* aspartokinase). Sequences of the two sets of primers used for gene amplification are *asp* (F 5′-GGT ATG ATT TCT ACA AAG CGA G-3′ and R 5′-ATA TAT CGT CGC GTG AAAGAC-3′) and *hipo* (F 5′-GAA GAG GGT TTG GGT GGT G-3′ and R 5′-AGC CGC ATA ATA ACT TAGCTTTG-3′). 

PCR was performed in final volume of 50 *μ*L mix containing 2.5 *μ*L of each deoxynucleoside triphosphate (10 mM), 2.5 *μ*L of MgCl_2_ (25 mM), 10 *μ*L of Buffer 5X DNA Taq polymerase, 0.4 *μ*L of Taq polymerase, 1.5 *μ*L of each primer *asp* (10 *μ*M), and 5 *μ*L of each primer *hipo* (10 *μ*M). Amplification reactions were carried out using thermal cycler (Gene Amp PCR system type 9700, Applied Biosystems, Villebon-sur-yvette, France) with the following program: an initial denaturation at 94°C for 15 min followed by 30 cycles of denaturation at 94°C for 1 min, annealing at 49°C for 1 min and polymerization at 72°C for 1 min. A final extension was performed at 72°C for 7 min. 

The amplification generated 735 bp and 500 bp DNA fragments corresponding, respectively, to *Campylobacter jejuni* and *Campylobacter coli*. The PCR products were stained with a 0.6% solution of ethidium bromide and were visualized under UV light after gel electrophoresis on 1.5% agarose. 

### 2.4. Antimicrobial Susceptibility Testing

Antimicrobial susceptibility testing was performed by disc diffusion method using Mueller-Hinton agar (Oxoid) supplemented with 5% defibrinated sheep blood, according to Clinical Laboratory Standards Institute (CLSI) guidelines [35]. Disks impregnated with antibiotics (Biomerieux, Marcy-l'Etoile, France) and their corresponding concentration are the followings: ciprofloxacin (CIP: 5 *μ*g); nalidixic acid (NA: 30 *μ*g); erythromycin (E: 15 *μ*g); gentamicin (GM: 10 UI); amoxicillin (AMX: 25 *μ*g). 

Briefly, well-isolated colonies of same morphological type were selected from an agar plate culture and transferred into 10 mL of sterile saline buffer (NaCl 0.9%). After homogenization, 2 mL of the mixture were flooded onto the surface of a Mueller-Hinton agar (Oxoid) containing 5% defibrinated sheep blood. The inoculum was allowed to dry for 5 min and antibiotic discs were placed on the plate. After 48 h of microaerobic incubation at 37°C, diameters of the inhibition zones were measured with calipers. *Escherichia coli* ATCC 25922 and *Staphylococcus aureus* ATCC 25923 were used as reference strains. The isolates were classified as sensitive, intermediate, and resistant according to the guidelines prepared by CLSI [35]. 

## 3. Results

### 3.1. Prevalence

Overall, 119 chickens ceaca were purchased from one commercial slaughter and cultured for thermophilic *Campylobacters. *From these 119 samples, 76 (63,8%) were positives and 43 (36.2%) were negatives to *Campylobacter* tests. Among the *Campylobacter* positives, 51.3% were *Campylobacter jejuni *and 48.7% were *Campylobacter coli*. 

### 3.2. Antimicrobial Susceptibility

The results of antimicrobial susceptibility testing for *Campylobacter* spp. isolated from chickens to nalidixic acid, ciprofloxacin, amoxicillin, erythromycin, and gentamicin are presented in Table 1.

For all isolates, antimicrobial resistance to nalidixic acid and ciprofloxacin was observed in 78.9% and 50%, respectively. 

Among the 39 isolates of *C. jejuni* 79.5%; 38.5%; 17.9%; 7.7% were resistant to nalidixic acid, ciprofloxacin, amoxicillin, erythromycin, and gentamicin, respectively.

Among the 37 isolates, 78.4%; 43.2%; 13.5%; 8.1% were resistant to nalidixic acid, ciprofloxacin, amoxicillin, and erythromycin, respectively. In this study all isolates of *C. coli* were sensitive to gentamicin. 

## 4. Discussion

Case-control studies of foodborne infection rates have estimated that 50 to 70% of *Campylobacter* illness is due to consumption of contaminated poultry and their products [9, 17, 36]. Several studies examined thermophilic *Campylobacter* in poultry, and the findings indicated prevalence ranges of the bacteria from 3% to 98% with *C. jejuni *or *C. coli *as the main isolates [36, 37].

Poultry and their products are commonly consumed in modern Ivoirians diets, but campylobacteriosis is not reported in Côte d'Ivoire up to now. Therefore, the primarily objective of this study was to determine prevalence of thermophilic species including *C. jejuni* and *C. coli, *in poultry in Abidjan. 

This work demonstrated high prevalence of thermophilic *Campylobacter* in the studied chickens (63.8%). This proportion is similar to the results obtained in previous study (66.6%) [28]. Among the *Campylobacter* isolates 51.3% were *C. jejuni* and 48.7% were *C. coli*. 

Such a high isolation rate of thermophilic *Campylobacter* in chickens has also been reported [37–42]. However, prevalence of *C. coli* in chicken is higher in this study than others findings [39, 42]. 

Poor hygiene and sanitation in poultry farms in Côte d'Ivoire could explain this high level of prevalence of *C. coli*. Indeed, most farms do not have security fence to prevent penetration of other animals including pigs, which are good carriers of *C. coli*. In some cases flocks of sheep or cattle and poultry take place at the same sites contributing like that to the contamination of chicken flocks with *C. coli*.

Antibiogram test indicated higher resistance of the microorganisms to ciprofloxacin and nalidixic acid. Recent studies reported that fluoroquinolone-resistant *Campylobacter* spp. rapidly emerged among poultry flocks [19, 24, 43, 44]. 

Alfredson and Korolik [45] advanced an hypothesis to explain this observation. They suggested that the use of enrofloxacin (derivates close to the fluoroquinolones used in human medicine) in animals flocks has probably exerted a selection pressure in animal reservoirs [22]. The absence of fluoroquinolone resistance of *Campylobacter* in Australia, country that has never used enrofloxacin, is a strong argument in favor of this hypothesis. 

Resistance to erythromycin and amoxicillin, two antibiotics widely used to treat illness in Côte d'Ivoire, is not negligible. However, rates of that resistance are lower compared to those obtained with the fluoroquinolones. 

These results are similar to previous observations [19, 20, 45–48]. The relatively high percentages of resistance to most antimicrobial agents tested in our study may be due to high usage of these agents as growth promoters or in animal treatment. In fact, in Cote d'Ivoire, as in most of developing countries [49], the use of antibiotics for humans and animals is relatively unrestricted.

Furthermore, no measures of hygiene are observed in both farms and in the process of slaughter which could cause contamination of poultry carcasses. Since campylobacteriosis is transmitted by consumption of contaminated food, preferably poultry, the presence of strains of antibiotic-resistant *Campylobacter* on chicken meat can be a real public health problem in Côte d'Ivoire. Indeed, the situation could deteriorate more rapidly in the study area, where there is an expansion of poultry farms with widespread and uncontrolled use of antibiotics. Therefore, surveillance of resistance pattern is necessary to guide rational use of antimicrobial agents in poultry farms. 

In conclusion, we reported the presence of high contamination in poultry in Côte d'Ivoire by thermophilic *Campylobacter* and high resistance to antimicrobials of the fluoroquinolone family. These results show the need to strengthen the implementation of specific control procedures to decrease the contamination of poultry meat by *Campylobacter* and the necessity to reduce using of antibiotics in poultry sector. This study also shows the need to establish an efficient system for the control of *Campylobacter* in chickens. 

## Figures and Tables

**Table 1:** Antimicrobial susceptibility of *Campylobacter jejuni* and *Campylobacter coli* isolated from chickens in 2009 in Abidjan (Cote d'Ivoire).

Campylobacter strains	Antibiotic disks				
	NA	CIP	AMX	E	GM
*Campylobacter *spp. (*n* = 76)					
R	60 (78.9%)	38 (50%)	9 (11.8)	10 (13.5%)	3 (3.9%)
I	7 (9.2%)	10 (13.2%)	14 (18.4%)	12 (15.8%)	0 (0%)
S	9 (11.9%)	28 (36.8%)	53 (69.8%)	54 (70.7%)	73 (96.1%)
*C. jejuni* (*n* = 39)					
R	31 (79.5%)	15 (38.5%)	4 (10.2%)	7 (17.9%)	3 (7.7%)
I	2 (12.8%)	6 (15.4%)	9 (23.1%)	8 (20.5%)	0 (0%)
S	6 (7.7%)	18 (46.1%)	26 (66.7%)	24 (61.6%)	36 (92.3%)
*C. coli* (*n* = 37)					
R	29 (78.4%)	16 (43.2%)	5 (13.5%)	3 (8.1%)	0 (0%)
I	2 (5.4%)	4 (10.8%)	5 (13.5%)	4 (10.8%)	0 (0%)
S	6 (16.2%)	17 (46%)	27 (73%)	30 (81.1%)	37 (100%)

S: sensitive, I: intermediate, R: resistant, NA: Nalidixic acid, CIP: Ciprofloxacin, AMX: Amoxicillin, E: Erythromycin, GM: Gentamicin.
